# The biomechanical and functional outcomes of fresh osteochondral allograft for the knee: A systematic review

**DOI:** 10.1016/j.jcot.2025.102983

**Published:** 2025-03-22

**Authors:** Stephanie Picioreanu, Leela Biant, Gwenllian Tawy

**Affiliations:** a Division of Medical Education, School of Medical Sciences, The University of Manchester, Manchester, UK; b Division of Cell Matrix Biology & Regenerative Medicine, School of Biological Sciences, The University of Manchester, Manchester, UK; c Department of Orthopaedics, Trafford General Hospital, Manchester University NHS Foundation Trust, Manchester, UK

**Keywords:** Fresh osteochondral allograft, FOCA, Knee, Range of motion, Biomechanics, Functional outcome

## Abstract

**Background:**

Fresh osteochondral allograft (FOCA) is a treatment option for osteochondral lesions of the knee which cause pain, locking, and joint instability. While FOCA aims to eliminate these mechanical symptoms, the biomechanical outcomes of the procedure remain poorly understood. This systematic review aimed to collate and interpret the available literature on the biomechanical outcomes of FOCA.

**Methods:**

Systematic searches were performed in three databases using the terms ‘(Knee OR knee joint) AND (FOCA OR fresh osteochondral allograft OR fresh OCA)’. Eligible studies contained objective biomechanical or functional outcomes, such as knee range of motion, strength, or parameters of gait. The National Institute of Health Quality Assessment Tool assessed study quality. Extracted data were synthesised in a spreadsheet and then a linear regression analysis was performed on the available range of motion data (p = 0.05). Data from a prior systematic review on the biomechanical outcomes of autologous chondrocyte implantation (ACI) were also included in this analysis to facilitate interpretation of the results. PROSPERO ID: CRD42024531998.

**Results:**

Eight studies with 54 participants met the inclusion criteria. On average, studies included 10 participants with a follow up range of 9–108 months.

Knee range of motion was reported to improve post-operatively in each study, and the post-operative range of motion was generally reported to be > 120°. A linear regression analysis showed no correlation between final range of motion and follow-up time (p value – 0.860; R^2^ - 0.019). These results did not differ statistically from the range of motion data reported in a prior review on ACI outcomes (F = 0.003; p = 0.874).

One study also reported an improvement in knee strength following FOCA, while two others commented on improvements to gait, although little numerical data was provided.

**Conclusion:**

The limited reporting of improvements to knee range of motion suggest that FOCA has the potential to improve patient quality of life through improved knee function. Linear regression analyses of data presented in this study and obtained from a prior report on the biomechanical outcomes of ACI suggest that the knee ranges of motion following both procedures are comparable. However, further research with larger patient cohorts and consistent methodologies are required to corroborate existing data. This knowledge is important for optimising outcomes via evidence-based rehabilitation programmes.

## 1 Introduction

The knee joint's articular cartilage and underlying subchondral bone are susceptible to defects as a consequence of direct trauma to the joint from a sporting incident or accident, osteochondritis dissecans or degenerative changes resulting from long-term repetitive loading of the knee.^1^^,^^2^ It is now well established that defects of the osteochondral unit can significantly impact an individual's quality of life, and if left untreated, predispose the patient to osteoarthritis of the knee.^1^, ^2^, ^3^

Common symptoms associated with osteochondral defects (OCD) include joint inflammation, pain, locking or catching, instability, and decreased range of motion.^4^, ^5^, ^6^ As a result, OCD can impair a patient's ability to perform activities of daily living and directly impact their working and social lives.^7^^,^^8^

Compromised cartilage tissue observed in OCD has limited capacity for self-repair.^4^^,^^9^^,^^10^ Consequently, tissue engineering techniques and cartilage repair surgeries have been developed to treat the injury and its symptoms.^5^ These procedures primarily aim to reduce pain and improve joint functionality.^11^

Fresh osteochondral allograft (FOCA) is one such surgical technique that is offered to patients with large (>2 cm^2^) symptomatic osteochondral lesions.^9^ According to NHS England's Clinical Commissioning Policy on FOCA, approximately 500 UK patients need treatment for a cartilaginous lesion in the knee >2 cm^2^ each year, and around 10 % of these individuals may be eligible for a FOCA.^12^ FOCA is primarily recommended where a primary defect is deep, or for salvage, where primary cartilage repair strategies have failed.

FOCA requires a graft of healthy cartilage from a recently deceased human donor. Thus, patient selection for the procedure is crucial. Once the donor tissue is harvested, the tissue is resized to match the size of the recipient's lesion and prepared for surgical transplantation.^13^ In successful cases, the donor's bone tissue heals to the recipient bone and is eventually replaced by recipient bone by creeping substitution. The chondrocytes remain the donors' cells for the life of the implantation. Thus, healing the OCD restores the joint's biomechanics.

Restoring joint biomechanics and preserving cartilage integrity is essential for preventing further damage to the osteochondral unit and delaying degenerative changes to the joint,^14^ A recent review by Familiari and colleagues reported that 47.8 % of patients across three studies had little to no radiographic evidence of osteoarthritis 9.3 years following FOCA.^15^^,^^16^ This suggests that FOCA can be effective at reducing the risk of onset and progression of osteoarthritis. This is supported by a study by Daud et al.*,* which reported that 10.13039/100030720FOCA delays total knee arthroplasty in young patients by 20 years. Their study also reported a 10-year graft survivorship rate of 73.3 % and an improvement in knee function for patients under 50 years old.^17^

Patient satisfaction is also high following FOCA. A study by Tírico et al.*,* reported an 88.1 % patient satisfaction rate in their study, which had a mean follow-up of 5.5 years. Their analyses showed that patients with patellar lesions and those who required a graft in two or more different locations within the knee were the least satisfied. The least satisfied patients were also shown to have significantly poorer patient reported outcome scores related to function, suggesting that biomechanical outcomes also play a role in patient satisfaction.^18^

While the current literature includes systematic reviews of the survivorship, clinical, and radiological outcomes of FOCA,^5^^,^^9^^,^^14^^,^^19^^,^^20^ there remains limited information on the biomechanical outcomes of the procedure. This is despite its known importance to clinical and patient-reported outcomes. This is not uncommon in joint preservation techniques, as evidenced in a recent review on the biomechanical outcomes of autologous chondrocyte implantation (ACI).^11^ Understanding how FOCA impacts functional outcomes is integral for clinician-patient interaction and patient autonomy. This knowledge is also important for tailoring post-operative rehabilitation programmes to optimise functional outcome. Successful restoration of joint biomechanics can reduce patients’ likelihood of developing osteoarthritis in the joint later in life^21^; thus, understanding how FOCA impacts knee function and biomechanics is academically and clinically important.

This systematic review aims to objectively quantify the known biomechanical and functional outcomes of FOCA and assess the efficacy of the procedure in improving the knee's function.

## 2 Methods

This systematic review was performed in accordance with the Preferred Reporting Items for Systematic Review and Meta-Analyses (PRISMA) statement. The protocol is registered on PROSPERO (ID: CRD42024531998).

### 2.1 Search strategy

A systematic search of the literature was conducted across three databases: Ovid MEDLINE, Embase, and Web of Science. The resources used within each database were searched from January 1, 1946 to March 29, 2024 and were accessed via the University of Manchester library. A final search was conducted in September 2024 to check for any new articles published since the original search was conducted. PROSPERO was also searched for ongoing, unpublished systematic reviews on the topic. The search terms utilised for this review in each database were ‘(Knee OR Knee joint) AND (FOCA OR fresh osteochondral allograft OR fresh OCA)’.

### 2.2 Eligibility

After conducting a primary search of the databases using the above-stated search terms, the identified publications were reviewed for inclusion by the authors. At first, the publications were reviewed by title and abstract only. Publications which did not meet the inclusion criteria were excluded, and the remaining publications were reviewed a second time. The second review involved review of the entire publication. Two individuals reviewed the studies for inclusion (SP, GT), and a third individual (LB) was available to provide further judgement where the two original reviewers disagreed on whether a study should be included or not.

Publications which met the following criteria were eligible for inclusion in this systematic review.

1. Studies that included patients who have undergone a type of FOCA procedure in the knee.
2. Studies that reported at least one quantitative biomechanical or functional outcome of FOCA (including, but not limited to: knee range of motion, outcomes of gait analysis such as 3D motion capture gait analysis, and knee strength assessments such as dynamometry).
3. The complete article was accessible.
4. The article was written in the English language.

The exclusion criteria were as follows.

1. Studies that included patients who have undergone any intervention for an OCD other than FOCA.
2. Studies which did not report any objective biomechanical variables.
3. Animal or cell studies.
4. Computerized or mathematical model studies.
5. Cadaveric studies.
6. Studies with data presented graphically or in an image only (i.e. data could not be extracted).

Studies in which FOCA was compared to other surgical techniques were eligible for inclusion in this systematic review on the condition that they also fulfilled the inclusion criteria. Dissertations and theses as well as conference proceedings and abstracts were also eligible for inclusion if they met the above criteria.

### 2.3 Quality assessment

The quality of papers that meet the eligibility criteria were assessed using the National Institute of Health Quality Assessment Tool for Case Series studies (Table 1). Each paper was assessed according to the following criteria: study design, clearly stated objectives, study population, participant recruitment, intervention description, outcome measures used, follow-up frequency, statistical analysis, results reported, and conflicts of interest. Each met criterion scored one point. Studies were assessed as ‘poor’ if they scored up to three points, ‘fair’ if they scored between four and six points, and a score of seven or above (out of nine) was deemed as ‘good’ quality. Two reviewers conducted the quality assessments (SP, GT). If the reviewers provided different scores within the same grade (Good, Fair, or Poor), then the average score was calculated and used. If the reviewers provided different scores that spanned different grades, then a third reviewer (LB) was invited to adjudicate.

**Table 1:** Design and study quality of all included papers.

Author and Year	Study Design	Level of Evidence	Study Quality	Declaration of Competing Interest
Bell (1994)	Case Series	Level IV	Fair	None Stated
Cusano (2018)	Case Report	Level IV	Good	None Stated
Daud (2021)	Case Report	Level IV	Fair	None Stated
Giannini (2013)	Case Report	Level IV	Fair	None Stated
Krettek (2017)	Case Report	Level IV	Good	None Stated
Lyon (2013)	Case Series	Level IV	Good	None Stated
McCulloch (2007)	Case series	Level IV	Good	None Stated
Vansadia (2016)	Case Report	Level IV	Good	None Stated

### 2.4 Data extraction

The primary data of interest in this review were the biomechanical outcomes. In this review, a biomechanical outcome is defined as any quantitative outcome related to the functionality of the knee, with the most common examples being knee range of motion in the sagittal plane (maximal extension subtracted from maximal flexion), assessments of knee strength, and quantitative variables of gait from gait analyses. The latter includes variables such as walking speed, cadence, distance walked during a set time, and more sophisticated data such as the average kinematics or kinetics of the knee during gait. Studies including only subjective measures and scoring systems were not included in this review.

Data on patient demographics, including average age, biological sex, average BMI, average size of defect, and follow up time for each of these studies were also extracted for analysis. Finally, the author and date of publication were also extracted for administrative purposes.

All data were extracted by one author (SP) and later reviewed by another author (GT) to ensure no errors were made in the data transfer process.

### 2.5 Data analysis

All data collected for the purpose of this review were compiled in Microsoft Excel. Each discreet variable was reported in its own column for easy analysis and interpretation of the results. Where enough data were available for a variable, the means, standard deviations, and reporting percentages were calculated using inbuilt functions within Excel. A linear regression analysis was also performed in GraphPad Prism (v 9.5.1, 2023; GraphPad Software Inc., California, USA) to investigate the relationship between the available range of motion data and follow-up time. Data from a previous review on the outcomes of ACI were also included in this analysis to assist our interpretation of the effectiveness of FOCA at restoring knee range of motion compared to another joint preservation technique.^11^ A test to determine whether the slopes and intercepts of both datasets was simultaneously performed.

## 3 Results

### 3.1 Search results

**Fig. 1:**
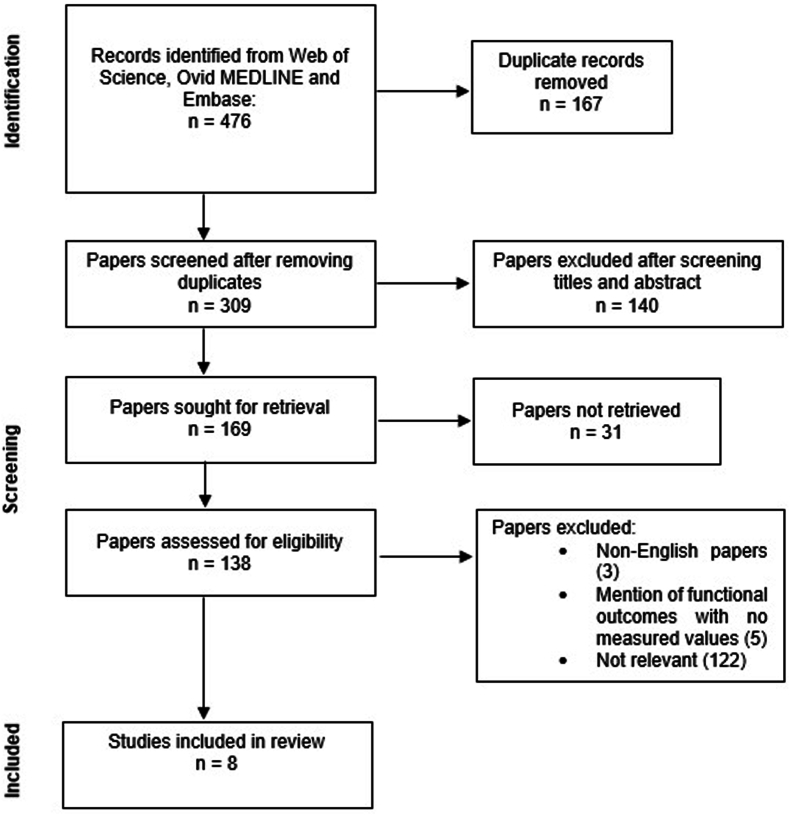
PRISMA diagram of the literature searching process.

Nine studies were initially identified as eligible for inclusion in this systematic review (Fig. 1). However, upon further investigation, one of the nine was later excluded because the relevant data from the patient described had been presented in another study that was already included in the review.^22^^,^^23^

The final literature search, performed six months after the original searches to ensure that no new eligible papers had been published while the review was conducted, identified one potential additional case report.^24^ On review of the full text, it was found that the individual in the case report had undergone a FOCA in combination with a high tibial osteotomy. As it was not possible to discern the individual impact of either procedure on the patient's biomechanical outcome the case report was not included in this review. Consequently, the final number of studies included in this review was 8.

### 3.2 Quality of included publications

The levels of evidence of the studies included in this review were low, as all were case reports or small case series. No case-control studies, cross-sectional studies, nor randomised controlled trials were identified; nor were there any existing systematic reviews on the subject (Table 1). The eligible publications generally scored well on the objectives, study population, participant recruitment, and intervention description. Marks were often lost for inadequate follow ups, unclear descriptions of the results and/or statistical analyses. None of the studies reported a conflict of interest, thus reducing the potential for bias towards the procedure outcomes.

### 3.3 Clinical population

Across all 8 studies, data were available for only 54 (85.7 %) of 63 consented study participants (Table 2). Males were more commonly included in the research studies than females (36M:27 F patients; 57.1 % Male), and the average reported age was 32.1 ± 12.5 years. Body mass index was only reported in two studies for two patients; one of whom was reportedly healthy and the other overweight.

**Table 2:** Patient characteristics for studies included in the review.

Author (Year)	Number of participants	Number of participants with available data	Mean Age (Years)	Male:Female	Mean BMI (kg/m^2^)	Mean Defect Size (cm^2^)	Type of procedure	Diagnosis	Mean Follow Up (months)
Bell (1994)^27^	16	13	33.5	6:10	–	–	FOCA	Giant cell tumour	108
Cusano (2018)^25^	1	1	25	0:1	27.3	10.70	FOCA	Osteonecrosis	12
Daud (2021)^26^	1	1	24	1:0	23.4	–	FOCA	Osteochondral defect	48
Giannini (2013)^23^	7	1	35.2	5:2	–	–	Bipolar FOCA	Osteoarthritis	60
Krettek (2017)^28^	1	1	58	0:1	–	–	FLOCSAT	Osteochondral defect	24
Lyon (2013)^29^	11	11	15.2	6:5	–	5.11	FOCA	Osteochondritis dissecans	24
McCulloch (2007)^30^	25	25	35	18:7	–	5.24	Prolonged FOCA	Osteochondral defect	35
Vansadia (2016)^31^	1	1	31	0:1	–	3.5	FOCA	Trochlear dysplasia	9

The patients were found to have a variety of diagnoses, which influenced the type of FOCA surgery undertaken (Table 2). Overall, three variations of the transplantation procedures were carried out – FOCA, bipolar FOCA, FLOCSAT (fresh large osteochondral shell allograft transplantation), and prolonged FOCA. In keeping with the variety of data presented in these studies, follow-up time ranged from 9 to 108 months (40.0 ± 32.4 months).

### 3.4 Findings

All eligible papers in this review reported range of motion as a functional outcome, however only 5 (62.5 %) reported pre-operative range of motion (Table 3). As such, it was not possible to calculate the average change in range of motion across all studies. Of the studies that reported both, range of motion was reported to increase post-operatively.

**Table 3:** Pre- and post-operative knee ranges of motion reported in included studies.

Author (Year)	Mean Preoperative ROM (°)	Mean Postoperative ROM (°)
Bell (1994)^27^	–	>90^a^
Cusano (2018)^25^	140	Complete
Daud (2021)^26^	110	130
Giannini (2013)^23^	–	80
Krettek (2017)^28^	120	130
Lyon (2013)^29^	–	≥125
McCulloch (2007)^30^	123	127
Vansadia (2016)^31^	130	140

a Data reported for a subset of the cohort only.

In a case study by Cusano et al.*,* the patient was reported to have achieved a range of motion of 90° six-weeks post-operatively with a stable knee (measured subjectively) under 0° and 30° valgus/varus stress. Six months post-operatively, the range of motion had been restored to ‘full’, although it was unclear whether this was greater or equal to the pre-operative range of motion of 140°, as no numerical data was provided.^25^

Daud and colleagues reported 5° fixed flexion with an average maximum flexion of 120° in their patient pre-operatively. Immediately post-operatively, the patient was reported to be able to fully extend the knee and achieve 90° flexion. This improved to a range of motion of 130° at 3 months. Seven years later, he was reported to have continual good stability and range of motion, although the quantitative values were not reported.^26^

Similarly, in Krettek's case study, the patient's pre-operative range of motion was 0-120°.^28^ Intra-operatively, this increased to 130°. Unfortunately, no further assessments were performed post-operatively. These findings were consistent with those reported by McCulloch and colleagues, who found an active pre-operative range of motion of 123° in the pre-operative knee and 129° in the contralateral knee; a clinically significant difference.^30^ Two-years post-operatively, a 4° improvement was reported in the operated knee, although the improvement was not statistically (p = 0.774) nor clinically significant. Similarities were also observed in Lyon's study on juvenile patients, where a minimum range of motion of 125° was reported 6-months following FOCA,^29^ and Vansadia et al.*,* case study where the patients' range of motion improved from 0 to 130° pre-operatively to 0-140° 9 months post-operatively.^31^

Other findings were not as positive. For example, Bell and colleagues reported only eight of their patients (61.5 %) were able to regain an active range of motion greater than 90° flexion at final follow-up. Across all patients in Bell's study, 10 (76.9 % of the study population) also had varus/valgus instability post-operatively; 6 with <10° instability, and 4 with >10° instability.^27^ Giannini et al.*,* reported full range of motion in their patient cohort at six months post-operatively, although failed to provide numerical data. Later in the study, 6 patients experienced a procedure failure, leaving only one patient to follow-up long-term. At the remaining patient's final follow-up, their range of motion was limited at 0-80°, although the patient was able to walk without a brace.^23^

**Fig. 2:**
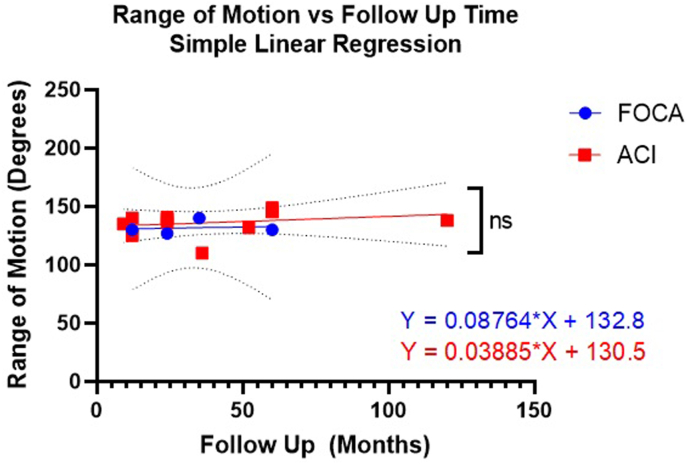
A simple linear regression of range of motion versus follow-up time for fresh osteochondral allograft (FOCA) and autologous chondrocyte implantation (ACI) procedures. Dotted lines show 99 % confidence intervals for both datasets. Note, the ACI data was obtained from a prior systematic review on the biomechanical outcomes of ACI.^11^

When reviewing the range of motion data collectively, it can be said that patients tend to present with near-to full extension and good range of motion. Post-operatively, knee range of motion appears to worsen in the short-term following FOCA, perhaps as part of the initial rehabilitation restrictions, but improve after a period of >6 months, at which point full extension and maximal flexion of >120° are generally reported. A linear regression analysis of the available data was carried out to determine whether a relationship exists between range and motion and follow-up time, but no significance was reported (Fig. 2; p value – 0.860; R^2^ - 0.019). Datasets which did not explicitly state the final range of motion were excluded, as was one outlying datapoint.^23^^,^^25^^,^^27^^,^^29^ Data from a prior review of ACI patients also showed no correlation between range of motion and follow-up time (Fig. 2; p-value – 0.408; R^2^ – 0.069). A test to assess whether the slopes of the FOCA and ACI datasets were significantly different found no significance (F = 0.003; p = 0.874, Fig. 2).

Five (62.5 %) of the included studies also described recording walking distance,^25^^,^^26^^,^^28^^,^^29^^,^^31^ although only two reported the results.^26^^,^^28^ Cusano and colleagues and Daud et al., reported their patients were largely immobile pre-operatively, with Daud's patient reportedly bedbound, and Cusano's patient only able to walk 50 feet.^25^^,^^26^ Unfortunately, no information was provided about post-operative walking ability. Lyon and colleagues also reported performing 0.8 km gait analyses, assessments of stair negotiation and walking aid requirements, but no quantitative data were reported in the paper.^29^ Similarly, Krettek reported that their patient could not mobilise without crutch or wheelchair support prior to their operation, and at 12-months, were able to walk for an unlimited distance without aids, but no quantitative data was provided.^28^ Bell's study reported that four of their patients continued to use a brace and additional walking aids when mobilising post-operatively, but again not quantitate data were reported.^27^

Finally, McCulloch's study (12.5 %) reported quadriceps size as a function of strength. They found that quadriceps size reduced from 47.2 cm to 46.2 cm 35 months post-operatively, although the reduction was not statistically significant (p = 0.987).^30^ The clinical impact of such a change remains unknown. Bell and colleagues also provided some insight into the quadriceps strength of their patient population, stating that ‘full quadriceps strength’ had returned in two patients following FOCA, while the remaining 5 patients continued to exhibit ‘minimal strength’.^27^ Again, no quantitative values were provided, nor were the methodologies used to measure and define strength reported. As with the gait data, no studies utilised standard biomechanical assessments of strength, such as dynamometry. As a result, too little information is currently available in the literature to understand the impact of FOCA on lower-limb strength.

## 4 Discussion

This systematic review has identified an existing lack of information and understanding of the biomechanical and functional outcomes of FOCA across all indications and populations eligible for the procedure. While the studies included in this review were generally of good quality, the levels of evidence were low.

Of the available literature, knee range of motion was the most frequently reported objective functional outcome. The limited literature suggests that FOCA may result in clinically significant improvements in range of motion post-operatively, as studies which assessed pre- and post-operative ROM reported an average improvement of 11°.^26^^,^^28^^,^^31^ This is greater than the minimum clinically important difference (MCID) commonly reported in the orthopaedic literature of ≥5° for surgical treatments and 3.8–6.4° for non-surgical treatments.^32^, ^33^, ^34^ Similar findings were reported in another of our systematic reviews on the functional and biomechanical outcomes of ACI, an alternative joint preservation surgery. The ACI review found patients’ ranges of motion increased by an average of 10° post-operatively.^11^ A linear regression analysis of the limited range of motion data available for these procedures is presented in this review. While no correlation was observed between range of motion and follow-up time for either procedure, there was no significant difference between the slopes of both datasets either. This suggests that the range of motion outcomes are comparable for ACI and FOCA. Collectively, these results show promise for joint preservation techniques like FOCA in improving range of motion. Care should be taken with the results of this regression analysis however, given the small sample sizes.

Importantly, 6 of the papers in this review reported a range of motion >120°, suggesting that the average FOCA patient has the available range of motion at the knee post-operatively to complete all activities of daily living, including some more demanding activities that require range of motion >110°, such as squatting. This is important, given the average FOCA patient in this review was in their early thirties, and therefore likely to be in employment and physically active. Nevertheless, it is important to caveat this information with the fact that range of motion data provide valuable insights into the kinds of activities a patient may be able to achieve post-operatively, but that the assessments are typically performed under static and not dynamic conditions. Thus, it is only possible to speculate the kinds of activities one could perform post-operatively with this data alone. Future research studies should explore the use of three-dimensional gait analyses to objectively assess patients’ spatio-temporal, kinematic and kinetic movement and gait patterns during dynamic activities. Such data would be clinically valuable for better setting patient expectations and preparing patients for their upcoming procedure and rehabilitation.

It should also be borne in mind that the findings of this report suggest that a period of rehabilitation for at least 3 months is likely necessary to improve range of motion >90°. This aligns with findings from a recent systematic review on the rehabilitative protocols adopted following FOCA; According to Stark and colleagues, patients were reported to return to full activity or sporting activity 6 months post-operatively, with return at 4 months being the earliest reported timeframe.^6^ Of the 62 eligible papers, Stark et al. reported that all rehabilitative protocols included weightbearing restrictions and guidelines on how range of motion should be progressed over time. However, the protocols varied significantly between papers. The authors also noted that objective functional assessments were rarely used to formulate goals for patients, or as means to monitor patient progress.^6^ This may explain why such little objective biomechanical data was identified by this review. Given the current lack of consensus on the most effective rehabilitation programme for FOCA, any existing local or national guidelines should be followed until an international consensus is agreed upon. This is particularly important when designing research studies, for methodological consistency.

Interestingly, the papers which reported the poorest range of motion post-operatively in this review were the oldest paper (1994) and the paper which investigated the use of FOCA for patients with more established osteoarthritis, suggesting that outcomes may have improved with improvements in technology and better patient selection.^23^^,^^27^ This theory is supported by recent research, which states that FOCA has evolved significantly over recent years. Examples of recent advances include improvements in graft storage methods, surgical techniques, and patient selection criteria.^35^ Specifically, optimized temperature conditions and low-oxygen environments have improved chondrocyte viability and extended storage durations without compromising the integrity of the extracellular matrix.^36^ New research findings are also changing our understanding of graft and recipient matching.^35^ For example, recent studies have reported no difference in failure rate, reoperation rate or outcomes in patients who received grafts from the lateral femoral hemicondyle to treat small lesions of the medial femoral condyle. This provides an opportunity to treat patients more efficiently and quickly, as lateral femoral hemicondyle grafts are more common than medial femoral hemicondyles. This is beneficial to the patient and for graft survivorship, as it means that some grafts no longer need to be stored until an exact condyle-match is found.^37^^,^^38^ Nevertheless, osseous integration remains paramount for treatment success.^35^ Consequently, t research investigating how to optimise osseous integration is ongoing. Some avenues with limited evidence include preparing the graft with pulsatile saline lavage,^39^ or combining the procedure with bone marrow aspirate concentrate.^40^, ^41^, ^42^ Collectively, these advances and research have resulted in improved outcomes for patients and broadened the criteria for FOCA to include older patients with focal cartilage defects, who were previously considered unsuitable for this procedure.

Returning to the results presented in this review, too little data was available on the spatiotemporal parameters of gait and knee strength post-operatively to be able to draw any conclusions of the impact of FOCA on these variables Importantly, no papers reported using ‘gold-standard’ 3D motion capture gait analysis or dynamometry to assess post-operative biomechanical outcomes of FOCA. This highlights the need for further research in this area. Previous studies investigating the functional outcomes of ACI reported significant improvements in walking distance at final follow-up, however all objectively quantified change by implementing the standardised 6-min walk test.^43^, ^44^, ^45^ As walking distance was not reported objectively in the papers included in this review, comparisons between the two patient populations cannot be made at this stage, nor can the MCID be calculated. Previous research has identified 74.3m as the MCID for the 6-min walk test, although this was calculated from a cohort of individuals 12-months post total knee arthroplasty.^46^ This figure may therefore not be appropriate to use to assess progress in patients undergoing joint preservation surgery.

Similarly, no previous studies on ACI patients are known to have used quadriceps size as a function of strength, limiting our ability to meaningfully compare this outcome between ACI and FOCA patient cohorts, nor draw any conclusions on the variable's MCID. Instead, previous studies in ACI have preferred to assess strength via straight leg raise and isokinetic strength tests.^43^, ^44^, ^45^^,^^47^, ^48^, ^49^, ^50^, ^51^ Nevertheless, quadriceps size is known to be reported in the literature as a function of strength following knee injury.^52^ A study conducted by Garcia et al., in 2020 investigated the correlation between quadriceps muscle size and knee extension strength in individuals who had undergone anterior cruciate ligament (ACL) reconstruction.^53^ This study used ultrasonography to measure the cross-sectional area of the quadriceps and dynamometry to assess knee extension strength. The correlated data suggested that muscle size, specifically its cross-sectional area, is a significant predictor of knee extension strength, highlighting the importance of muscle hypertrophy in functional recovery.^53^ This relationship was also observed by Mitchell et al., in 2012. Their study highlighted that in aging populations, muscle quality (e.g., fiber composition and neuromuscular efficiency) influences strength more significantly than size alone. These findings display the importance of assessing both muscle size and quality in assessing strength, particularly in clinical and aging contexts.^52^ Further research is also recommended to investigate the association between quadriceps size and muscle strength in the clinical context, to facilitate data interpretation.

This study is limited by the fact that a meta-analysis of the data could not be performed, as none of the studies included a comparative cohort. It is therefore difficult at present to draw meaningful conclusions on the effectiveness of FOCA at restoring knee biomechanics and function compared to other joint preservation techniques. McCulloch and colleagues explained that it was not appropriate to incorporate a comparative cohort into their methodology, as it was deemed unethical to withhold treatment from symptomatic patients who met the criteria for FOCA.^30^ Other studies have overcome this ethical dilemma by comparing the outcomes of FOCA to ACI. While both FOCA and ACI are joint preserving treatments, randomising patients to FOCA or ACI is not always appropriate. For example, the UK's National Health Service state that FOCA should be performed in knees with lesions >2 cm^2^ which show significant diseased bone or bone loss, while ACI is most appropriate for lesions >2 cm^2^ with minimal bone abnormality.^12^ ACI is only indicated for patients who fit the former criteria if waiting for a FOCA is believed to lead to patient deterioration.

Studies which have compared the outcomes of FOCA to ACI have reported conflicting findings. One study reported the Knee injury and Osteoarthritis Outcome Score for Joint Replacement (KOOS-JR) at 2 years to be significantly greater following ACI when compared to FOCA,^54^ while two other studies reported similar outcomes in the KOOS-JR and International Knee Documentation Committee (IKDC) scores 2- and 6.7-years post-operatively.^55^^,^^56^ Similarities between both cohorts have also been reported in Tegner and Lysholm scores 2-years post-operatively.^56^

Conflicting findings have also been reported between FOCA and ACI with regards to revision rates. Although Hanna et al.*,* and Riff et al.*,* reported similar revision rates at 2 years,^54^^,^^56^ Matthews and colleagues reported lower revision rates for ACI for multifocal and condylar lesions at 6.7 years.^55^ With regards to reoperation rates, Sochaki and Riff reported higher reoperation rates for ACI than FOCA at 2-years, but these differences were only significant in Sochaki's study (67.6 % vs. 40.4 %).^56^^,^^57^ One recent systematic review and meta-analysis has also included matrix-induced ACI (MACI) and osteochondral autograft transplantation (OAT) in their investigations, showing that all four procedures result in improved pain and functional outcomes as reported by Lysholm, IKDC, Tegner and Visual Analogue Score.^58^ The patient acceptable symptom state (PASS) was achieved for all procedures according to the Lysholm and Tenger scores. Only OAT achieved PASS for the IKDC. However, the MCID was achieved for all outcomes across all procedures.^58^ Nevertheless, the authors conclude that patient history and defect characteristics must still be considered when determining the most appropriate treatment pathway for the patient.

Existing studies comparing the outcomes of FOCA to other joint preserving surgeries provide a flavour for the complexity in analysing the emerging data in this field and highlight the need for further research. Importantly, none of the studies which have compared the outcomes of FOCA and ACI reported any biomechanical outcomes. Our limited linear regression analysis of the ranges of motion reported following FOCA and ACI provide some insight, but more research is required to improve our understanding of the impact of these procedures on biomechanical outcomes.

A further limitation of this review is the overall quality of the collective dataset. The dataset is poor due to the varied methodologies and patient populations reported. Few patients were included in the studies, and the follow-up periods ranged from 9 to 108 months. Arguably, the datasets are thus incomparable. Moreover, a variety of methods were utilised to report the same variable. Most notably, range of motion was not reported consistently across the papers, making cross-study statistical analyses impossible and drawing conclusions from the available data difficult. This was also observed in our previous review on the functional outcomes of ACI, highlighting the need for consistent reporting in clinical studies in the field of joint preservation.^11^ We would recommend that future researchers report the maximum flexion and extension values at baseline and post-operatively for clarity. In this review, only two papers clearly described whether the range of motion assessments were performed actively or passively.^27^^,^^30^ Although one additional study reported assessing range of motion passively and actively, only one figure was reported in the manuscript.^29^ To facilitate data interpretation, passive and active range of motion should be reported where possible; Where only one assessment can be performed, the type of assessment should be clearly noted in the publication. These assessments should also be used in combination with other assessments of function and mobility, for a better-rounded view of patients’ biomechanical outcomes.

## 5 Conclusion

The studies included in this systematic review reported improvements in patient's functionality following FOCA, particularly with regards to knee range of motion. A linear regression analysis of the data presented in this study and from a prior report on the biomechanical outcomes of ACI suggest that the post-operative knee ranges of motion are comparable in both cohorts. However, the current available literature is too diverse and the sample sizes too small to draw substantial conclusions on the impact of FOCA on short- and long-term knee function and its biomechanical performance compared to other joint preservation techniques. Further research is necessary to better understand how FOCA impacts knee biomechanics and mobility, particularly during dynamic activities of daily living. This knowledge will be important for informing future rehabilitation programmes to optimise patient outcomes, and subsequently adequately assess the procedure's long-term performance and effectiveness at reducing the risk of osteoarthritis onset and/or progression.

## CRediT authorship contribution statement

**Stephanie Picioreanu:** Data curation, Formal analysis, Investigation, Methodology, Validation, Visualization, Writing – original draft, Writing – review & editing. **Leela Biant:** Supervision, Project administration, Writing – review & editing. **Gwenllian Tawy:** Conceptualization, Data curation, Formal analysis, Investigation, Methodology, Project administration, Supervision, Validation, Writing – original draft, Writing – review & editing.

## Informed consent statement

It was not necessary to obtain informed consent for this study, as it did not involve participants.

## Guardian/patient's consent

Guardian/Patient consent was not required for this study, as it was a systematic review of the literature.

## Data availability statement

As this study is a systematic review, all data included in the study may be accessed via the original papers included in this review.

## Ethics approval statement

Ethical approval was not required for this study, as it was a systematic review of the literature.

## Ethics approval statement

Ethical approval was not required for this study, as it was a systematic review of the literature.

## Funding statement

This research did not receive any specific grant from funding agencies in the public, commercial, or not-for-profit sectors. The study was performed as part of an educational project towards an MBChB degree at the University of Manchester.

## Declaration of competing interest

The authors declare that they have no known competing financial interests or personal relationships that could have appeared to influence the work reported in this paper.
